# Purification, structural analysis, and stability of antioxidant peptides from purple wheat bran

**DOI:** 10.1186/s13065-020-00708-z

**Published:** 2020-10-03

**Authors:** Yan Zhao, Qi Zhao, Qingyu Lu

**Affiliations:** 1 National Engineering Laboratory of Wheat & Corn Further Processing, Henan University of Technology, Zhengzhou, 450001 China; 2 College of Food Science and Technology, Henan University of Technology, Zhengzhou, 450001 China

**Keywords:** Purple wheat bran, Antioxidant peptide, Purification, Structural analysis, Stability

## Abstract

Protein derived from purple wheat bran was hydrolyzed sequentially using alcalase proteases for the production of antioxidant peptides. Purple wheat bran protein (PWBP) hydrolysates were fractionated using size-exclusion (G-25) and ion-exchange chromatography methods to identify the structure of antioxidant peptides. The free radical scavenging activity of peptides purified from PWBP hydrolysates was evaluated using superoxide anion radical-scavenging activity and determination assays of Trolox equivalent antioxidant capacity (TEAC). Results demonstrated that purple wheat bran peptide F4-4 exhibited the highest antioxidant activity among other hydrolysates. F4-4 was further identified as Cys-Gly-Phe-Pro-Gly-His-Cys, Gln-Ala-Cys, Arg-Asn-Phe, Ser-Ser-Cys, and Trp-Phe by high performance liquid chromatography (HPLC) spectrometer coupled with Orbitrap Elite™ mass spectrometer (LC–MS/MS). Antioxidant peptides 2 and 4 showed improved stability when the temperature was lower than 80 °C. These peptides also demonstrated good digestive stability in vitro system by simulating gastrointestinal digestion.

## Introduction

Antioxidants are substances that counteract the oxidation reaction caused by free radicals. Antioxidants are used to extend the shelf life of foods because they reduce lipid oxidation [1]. Synthetic antioxidants are commonly used in the food industry [2]. The most widely used synthetic antioxidants are butylated hydroxy anisole (BHA), butylated hydroxytoluene (BHT), and tert-butylhydroquinone (TBHQ). However, the use of synthetic antioxidants is strictly regulated because of their adverse effects to human health [3]. Studies have shown that these synthetic antioxidants can have varying degrees of toxicity in humans and affect the liver, spleen, and lung [4–6]. Therefore, the use of these synthetic chemical substances has been strictly limited [7]. Currently, considerable attention has been provided to natural antioxidants due to their high efficacy and little or no side effects compared with synthetic antioxidants [8, 9].

Peptides derived from natural sources are natural antioxidants. These antioxidants have been the focus of research interest because of their potential health benefits associated with low molecular weight, low cost, high activity, functional properties, and safety [10, 11]. Many studies have produced bioactive peptides from plant protein sources. Thus, natural antioxidants from grains, vegetables, and plant substrates are crucial in protecting the body from reactive oxygen species (ROS) damage. Bioactive peptides can act as inhibitors of lipid peroxidation to scavenge free radicals directly [11] and prevent damages associated with oxidative stress in humans with regular consumption [9]. Most antioxidant peptides are small in size (less than 1 kDa, typically bioactive peptides), have 2 to 20 amino acid residues per chain, and usually contain a high proportion of hydrophobic amino acids [9]. Previous studies have shown that antioxidative peptides can be purified from several plant protein hydrolysates, such as soybean [12], rapeseed [11], wheat[13], and rice bran [14]. Several antioxidant properties of these peptides, including their capability to inactivate ROS, scavenge free radicals, and chelate pro-oxidative transition metals [15, 16], have been described.

Purple wheat is one of the wheat varieties. Most studies on purple wheat bran investigated its anthocyanins because of its color [11, 17]. However, these studies ignored the importance of purple wheat bran as a protein resource and its capability to extract antioxidant peptides. In the present study, the hydrolysis of purple wheat bran by a protease to produce peptides with high antioxidant activities was studied. Furthermore, the antioxidant peptides were isolated and purified, sequenced, and studied for stability.

## Materials and methods

### Materials

Purple wheat (*Triticum aestivum L.*) of the cultivar Shannong Purple Wheat One were harvested in August, 2018 (base on the GB 1351–2008) at maturity from Shangdong, China. Purple wheat bran protein (PWBP) was prepared from purple wheat bran. Alcalase (2 × 105 U/g), flavourzyme (3 × 104 U/g), papain (8 × 105 U/g), neutral enzyme (3 × 104 U/g), and trypsin (2.5 × 105 U/g) were purchased from Beijing Solarbio Science &Technology Co., Ltd. (Beijing, China) DETA-Sepharose FF and Sephadex G-25 were purchased from Beijing Solarbio Science &Technology Co., Ltd. (Beijing, China).

### Enzymatic hydrolysis of PWBP

Pretreated PWBP (2 g) was dissolved in 100 mL of distilled water and then hydrolyzed with alcalase (50 °C, pH 8.0), flavourzyme (53 °C, pH 6.4), papain (50 °C, pH 7.0), neutral enzyme (39 °C, pH 7.0), and trypsin (45 °C, pH 8.0) respectively under their optimal conditions as recommended by the manufacturer. These five enzymes have a hydrolysis time of 60 min and an enzyme addition of 14, 000 U/g, and then heated in 100 °C water bath for 10 min to inactivate the enzymes. Degree of hydrolysis (DH), peptide yield and the TEAC of PWBP hydrolysates were determined. The best enzyme was selected and hydrolyzed for 0.5‒3 h. DH, peptide yield and TEAC were determined every 0.5 h. The DH and peptide yield were determined by pH–stat method and Lowry method [18, 19]. TEAC was determined as described in 2.4.2.

### Purification of antioxidant peptides from PWBP hydrolysates

#### Sephadexed-G25 chromatography

PWBP hydrolysates, which were prepared by alcalase as chosen in 2.2, applied onto a Sephadexed-G25 column (1.5 × 75 cm) and eluted with deionized water at a flow rate of 1.6 mL/min and monitored at 280 nm. The active fractions were collected, lyophilized and determined their antioxidant activities using the methods described in 2.4. The best strong antioxidant fraction was selected and used as material in next chromatography separation step.

#### Ion exchange chromatography

The fraction exhibiting antioxidant activity by 2.3.1 was loaded onto a DEAE-Sepharose Fast Flow column (1.6 × 30 cm), which was earlier equilibrated with 20 mM Tris–HCl buffer (pH 8.0) and eluted with a linear gradient of NaCl (0–1.0 mol/mL) in the same buffer at a flow rate of 0.6 mL/min. Each fraction separated and purified by ion exchange chromatography was monitored at 280 nm, collected at a volume of 3 mL and concentrated using a vacuum rotary evaporator. Antioxidant activities of separated fractions were investigated using the methods described in 2.4.

### Determination of antioxidant activities

#### Superoxide anion radical scavenging activity assay

Superoxide anion radical scavenging activity assay was measured using the pyrogallol autoxidation method with some modifications [20]. Mix 1.6 mL of sample with an equal volume of 50 mM Tris–HCl buffer (pH 8.3). The mixture was incubated in a bath for 20 min, then 0.8 mL of 1.5 mM pyrogallic acid was added. Absorbance of the mixture was measured at 320 nm every 30 s for 5 min. For blank, 1.6 mL of Tris–HCl buffer was used instead of sample. Ascorbic acid and GSH were used as positive controls. Superoxide anion radical scavenging activity was calculated as: $$\mathrm{O}2-\mathrm{ clearance rate }\left(\mathrm{\%}\right)=\frac{{1-\mathrm{\Delta OD}}_{\mathrm{S}}}{{\mathrm{\Delta OD}}_{\mathrm{b}}}\times 100\mathrm{\%},$$$$\mathrm{Where},{\mathrm{ \Delta OD}}_{\mathrm{S}} \mathrm{and} {\mathrm{\Delta OD}}_{\mathrm{b}}$$ represented slope of absorbance line for sample and slope of absorbance line for blank.

#### Determination of Trolox equivalent (TE) antioxidant capacity (TEAC)

The experiment referred to methods of Costa and Xing and made appropriate modifications [21, 22]. This method used Trolox as the standard antioxidant and all reagents were prepared in 50 mmol/L phosphate buffered solution (pH 7.4). The specific operation was as follows: PBS (50 µL) solution (blank group), different concentrations of Trolox solution (standard control group) and sample solution to be tested (sample group) were added to a transparent 96-well microplate, and then 150 µL ABTS^+^· was quickly added. The solution was shaken at 30 °C for 30 min and its absorbance was measured at 734 nm using a microplate scanning spectrophotometer (Multiskan FC, Thermo Fisher Scientific (Shanghai), China). ABTS radical scavenging activity was calculated as

$$\mathrm{ABTS}+\cdot \mathrm{ clearance rate }\left(\mathrm{\%}\right)=\left(1-\frac{{\mathrm{A}}_{\mathrm{Sample}}}{{\mathrm{A}}_{\mathrm{Blank}}}\right)\times 100$$

where, ${\mathrm{A}}_{\mathrm{Sample}}$ and ${\mathrm{A}}_{\mathrm{Blank}}$ referred to the absorbance of sample group and blank group, respectively.

### Identification of the antioxidative peptides by LC–MS/MS

For identification of sequences, the purified peptides by 2.3 were subjected to a capillary high performance liquid chromatography (HPLC) spectrometer coupled with Orbitrap Elite™ mass spectrometer source at Biotech-Pack Scientific Co., Ltd. (Beijing, China). Following molecular mass determination, the peptide was automatically selected for fragmentation, and sequence information was obtained by tandem mass spectroscopy (MS/MS) analysis. Data were processed using Xcalibur software (Thermo Scientific MS software) and the Maxquant (1.6.2.10) auxiliary tool within the protein database restricted to wheat entries.

### Synthesis of antioxidant peptides

The identified purple wheat bran antioxidant peptides were synthesized by the solid phase method using 9-fluorenylmethoxycarbonyl (FMOC) amino acids method at Biotech-Pack Scientific Co., Ltd. (Beijing, China). The purity of the synthesized peptides was greater than 90%.

### Stability study

#### Temperature stability

The antioxidant activities of purple wheat bran antioxidant peptides at different passivation temperatures for 60 min were studied. The detection method of antioxidant activities was shown in 2.4. Before the antioxidant activities were measured, the samples allowed to cool to room temperature (25 °C) and pH was adjusted to 7.0.

### In vitro* digestion stability*

According to the main components of human gastrointestinal digestive juice and digestive environment, a human gastrointestinal digestion simulation system was established to study the antioxidant activity changes of peptides after digestion by human gastrointestinal digestion simulation system. The method was according to the previously reported by Jang et al. [23].

### Statistical analyses

All experiments were carried out in triplicate, and all results were expressed as the mean ± standard deviation. Results were analyzed by one-way analysis of variance with SPSS (Version 20, IBM Corp., Armonk, NY). Means followed by different letters were significantly different according to Duncan’s multiple range test at *P* < 0.05 level.

## Results and discussion

### Enzymatic hydrolysis of PWBP by various proteases

The degree of hydrolysis (DH) of purple wheat bran protein (PWBP), which was enzymatically hydrolyzed by alcalase, flavourzyme, papain, neutral enzyme, and trypsin, as well as the peptide yield and Trolox equivalent antioxidant capacity (TEAC) of PWBP hydrolysates, were investigated in this study (Table 1). Results showed that the peptide yield and the TEAC of PWBP hydrolysates prepared from alcalase were remarkably higher than those of the other enzymes’ hydrolysates. The DH of PWBP hydrolysates by flavourzyme was substantially higher than those of other enzymes, their peptide yield and TEAC were lower than those of alcalase. Therefore, alcalase was selected to prepare the hydrolysates of PWBP.

**Table 1:** The DH of PWBP enzymatic hydrolysis by five enzymes, and peptide yield and TEAC of PWBP hydrolysates

	Alcalase	Flavourzyme	Papain	Netural enzyme	Trypsin
DH (%)	4.88 ± 1.27^b^	6.42 ± 0.25^a^	3.05 ± 0.36^c^	3.45 ± 0.53^bc^	3.51 ± 0.51^bc^
Peptide yield (%)	43.67 ± 8.86^a^	9.44 ± 1.35^c^	20.06 ± 1.84^b^	27.74 ± 0.51^b^	23.61 ± 2.23^b^
TEAC (µmol TE/g)	917.11 ± 5.89^a^	428.02 ± 44.00^b^	344.34 ± 6.21^bc^	206.33 ± 5.33^c^	783.43 ± 3.71^b^

Data expressed as means ± SD (*n* = 3). Values in the same row followed by different letters were significantly different at* P* < 0.05

**Fig. 1.**
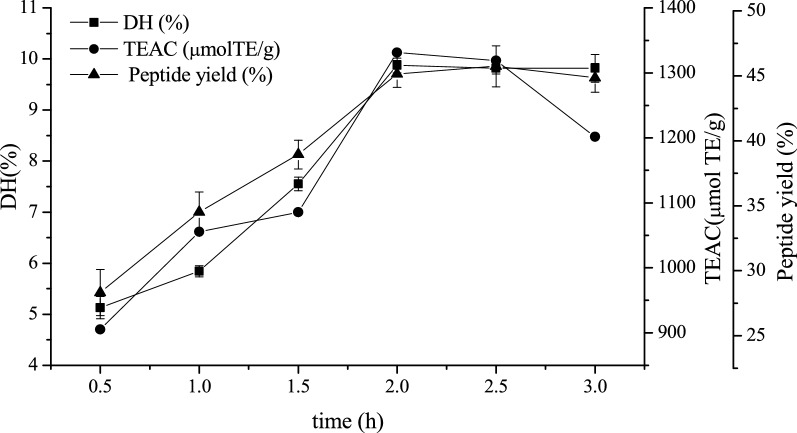
Changes in DH of PWBP enzymatic hydrolysis by Alcalase, and peptide yield and TEAC of PWBP hydrolysates in different times. Data expressed as means ± SD (n = 3)

Moreover, the DH of PWBP and the peptide yield of PWBP hydrolysates prepared by alcalase increased in 0.5–2 h and remained stable for the remaining 2–3 h (Fig. 1). This result was consistent with that of the general proteolytic curve studied by Pedro Valencia et al. [24]. The TEAC of PWBP hydrolysates by alcalase increased in 0.5–2 h and gradually decreased with the increase in time (Fig. 1). The TEAC results might be due to the cleavage of most peptide bonds during hydrolysis [25]. The antioxidant activity of wheat bran peptide decreased when the DH of the substrate exceeded the optimal DH of the wheat bran peptide. DH, peptide yield, and TEAC were the highest at 9.88%, 45.70%, and 1331.10 µmol TE/g, respectively, when the PWBP hydrolysates were prepared by alcalase for 2 h. Therefore, hydrolysis time with alcalase in the subsequent experiments was 2 h.

### Purification of antioxidant peptides from PWBP hydrolysates

**Fig. 2.**
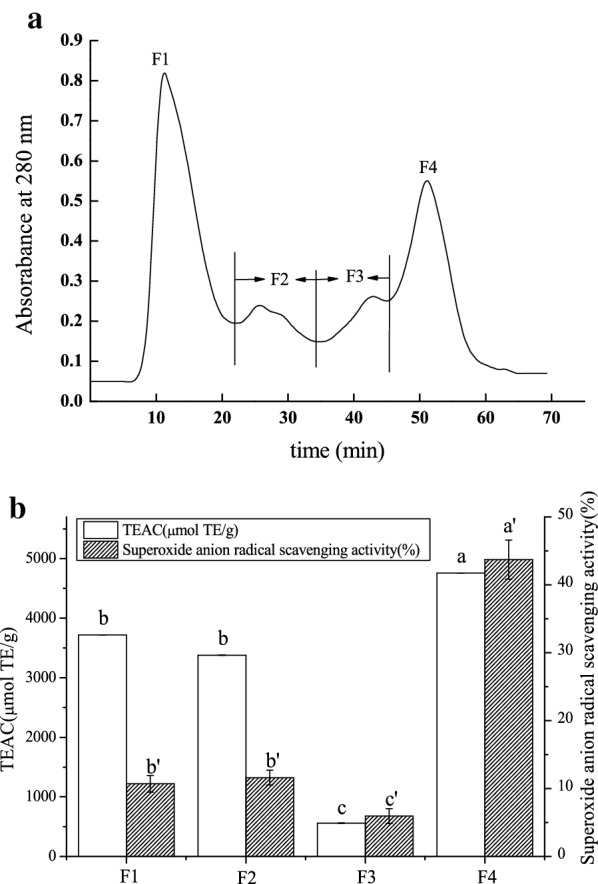
The profiles of PWBP hydrolysates using size exclusion chromatogram on a Sephadex G-25 column (**a**) and superoxide anion radical scavenging activity and TEAC of various fractions at peptide concentration of 5 mg/mL. Means (n = 3) followed by the same letter were not significantly different according to Duncan’s multiple range test at P < 0.05 level

Purple wheat bran protein hydrolysates were initially separated by size-exclusion chromatography through a Sephadex G-25. The profiles of PWBP hydrolysates and the antioxidant activity of each component are shown in Fig. 2. Purple wheat bran protein hydrolysates were fractionated into four fractions (F1–F4). Results show that fraction 4 (F4) possesses the highest antioxidant capacity. Figure 2b reveals that the superoxide anion radical scavenging activity and TEAC of F4 were 43.72% ± 2.9% and 4760 ± 1.1 µmol TE/g, respectively (Fig. 2b).

**Fig. 3.**
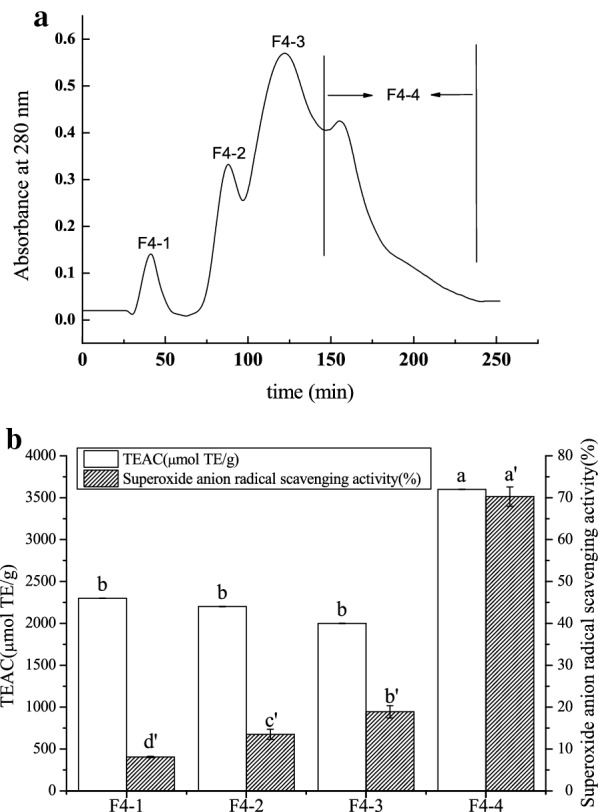
Ion exchange chromatogram of F4 on a DEAE-Sepharose Fast Flow column (**a**) and superoxide radical scavenging activity and TEAC of various fractions at peptide concentration of 5 mg/mL (**b**). Means (n = 3) followed by the same letter were not significantly different according to Duncan’s multiple range test at P < 0.05 level

F4 was further subjected to ion-exchange chromatography on a DEAE-Sepharose Fast Flow column and separated into F4-1, F4-2, F4-3, and F4-4 (Fig. 3a). The F4-4 exhibited superoxide anion radical scavenging activity and TEAC of 70.27% and 3600 µmol TE/g, respectively, which were remarkably higher than those of the other fractions (Fig. 3b). Therefore, the F4-4 fraction was collected for determining amino acid sequences.

### Sequence identification

**Fig. 4.**
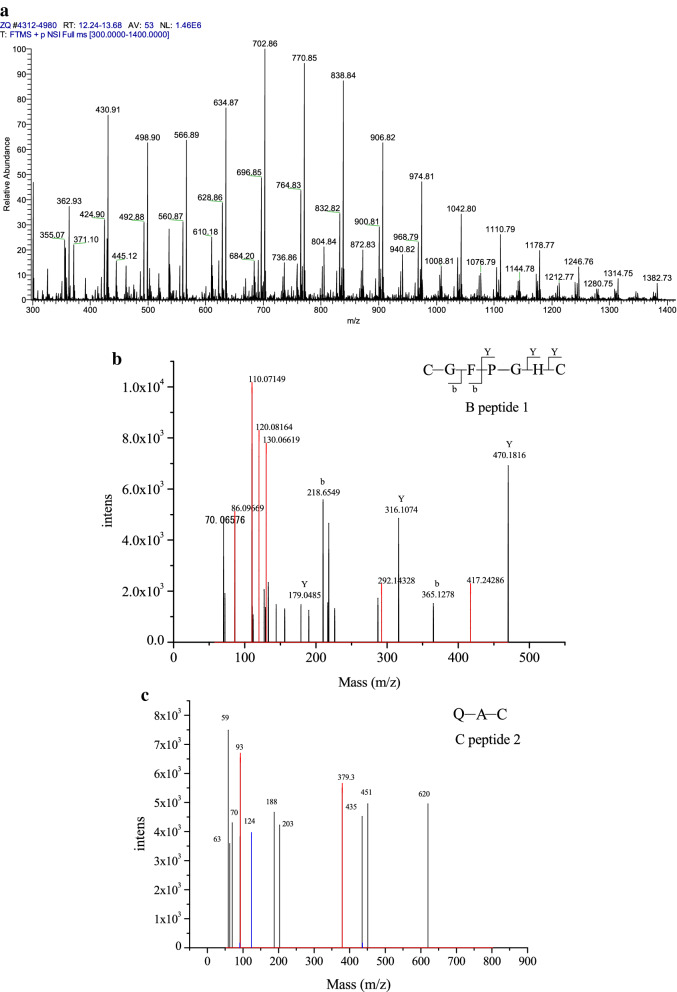

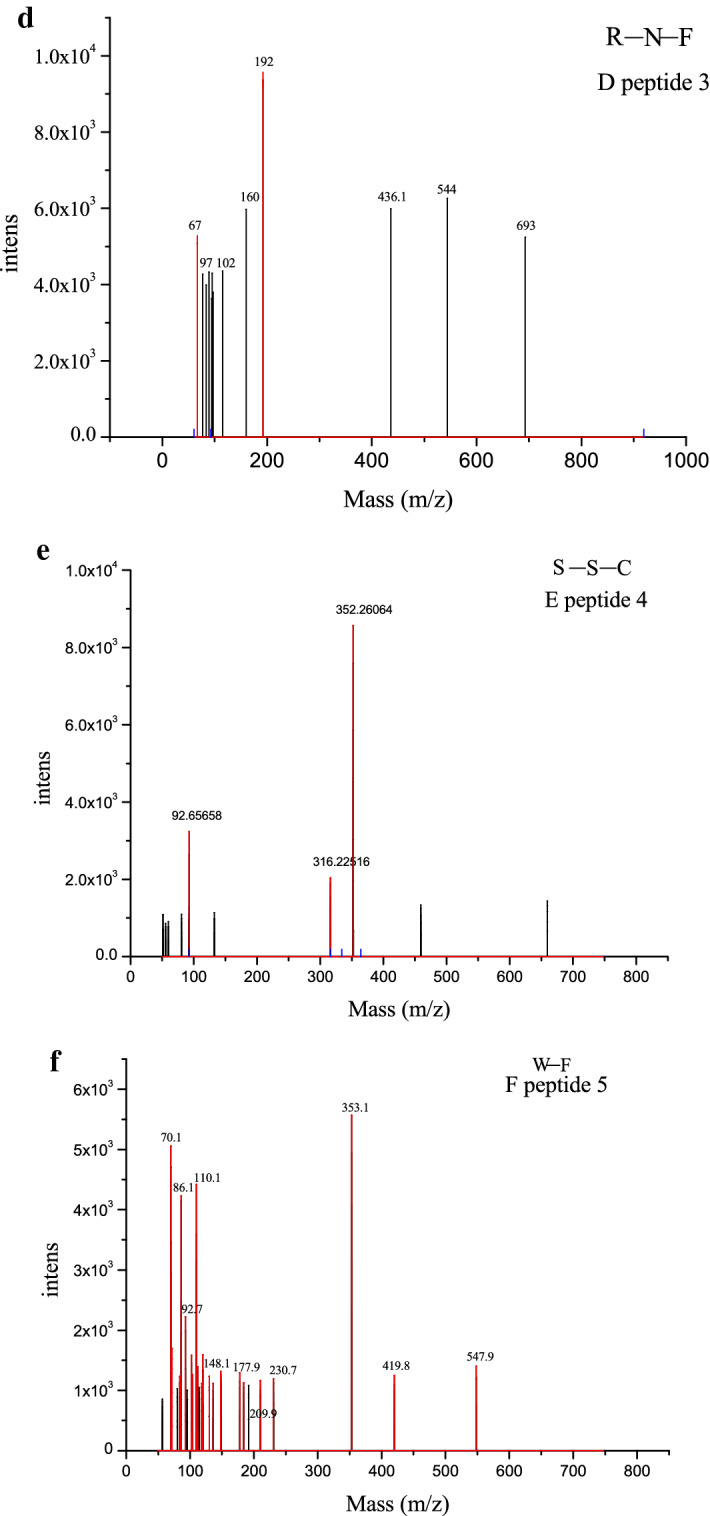
Amino acid sequence identification by LC/MS. The mass spectrum of **a**. The MS/MS spectrum of peptide 1 (**b**, CGFPGHC). The MS/MS spectrum of peptide 2 (**c**, QAC). The MS/MS spectrum of peptide 3 (**d**, RNF). The MS/MS spectrum of peptide 4 (**e**, SSC). The MS/MS spectrum of peptide 5 (**f**, WF)

The peptide sequence is of considerable importance in determining the antioxidant activity of active peptides [16, 26]. Thus, identifying the specific features of the sequences of antioxidant peptides are necessary. The mass spectrum is shown in Fig. 4a, and the tandem mass spectra of peptide 1–peptide 5 are shown in Fig. 4b–f. The matching result provided the following putative amino acid sequences: Cys-Gly-Phe-Pro-Gly-His-Cys, Gln-Ala-Cys, Arg-Asn-Phe, Ser-Ser-Cys, and Trp-Phe.

Kim et al. and Qian et al. [27, 28] studied the majority of antioxidant peptides and showed that those with low molecular weight are efficient antioxidants. Moreover, they found that bioactive peptides usually contain less than 20 amino acid residues per molecule and can cross the intestinal barrier and exert biological effects. In the present study, all five antioxidant peptides had low molecular weights (< 600 Da), which are consistent with the results of Kim et al. and Qian et al. Therefore, the five peptides identified in this experiment had improved antioxidant activity.

Almost all bioactive peptides containing Tyr, Leu, Ala, Ile, Val, Lys, Phe, Cys, Met, and His exhibit high antioxidant activities [9]. Another study also indicated that antioxidative peptides often contain hydrophobic amino acid residues, such as Pro, Tyr, Trp, Met, Gln, and Ala, which are generally considered antioxidants in some cases [1, 29, 30]. The present results show the following: peptide 1 (Fig. 4b) contains Cys, Phe, Pro, and His; peptide 2 (Fig. 4c) contains Gln, Ala, and Cys; peptide 3 (Fig. 4d) contains Phe; peptide 4 (Fig. 4e) contains Cys; and peptide 5 (Fig. 4f) contains Trp and Phe. Therefore, the antioxidant activity of peptides 1 to 5 isolated from purple wheat bran is also related to the existence of the aforementioned amino acids. This finding is consistent with that of Kou et al. [16].

Qian [28] found that His, Trp, and Phe with aromatic residues can stabilize ROS through direct electron transfer. Moreover, Phe, which positively acts as a direct radical scavenger [31], was also present in the antioxidative peptide sequences of peptides 1 (Fig. 4b), 3 (Fig. 4e), and 5 (Fig. 4f) in the present study. Phe could also be partly responsible for the antioxidant activity of these peptides.

Sequence analysis indicated that peptides 1(Fig. 4b), 2 (Fig. 4c), and 4 (Fig. 4e) contained hydrophobic amino acids, such as Cys. Qian et al. [28] found that Cys is crucial for antioxidant action because it can directly interact with radicals. Thus, the antioxidant activity of peptides 1, 2, and 4 are assumed to be related to Cys. Previous research also confirmed this finding. Harman et al. [32] reported that the thiol group of Cys is crucial in protecting cells and cellular biomolecules from oxidative stress.

Based on these results, peptides with low molecular weight and good antioxidant activities were obtained from PWBP by enzymatic hydrolysis to enhance the utilization value of PWBP and avoid resource waste.

### Evaluation of antioxidant activities of synthesized peptides

**Fig. 5.**
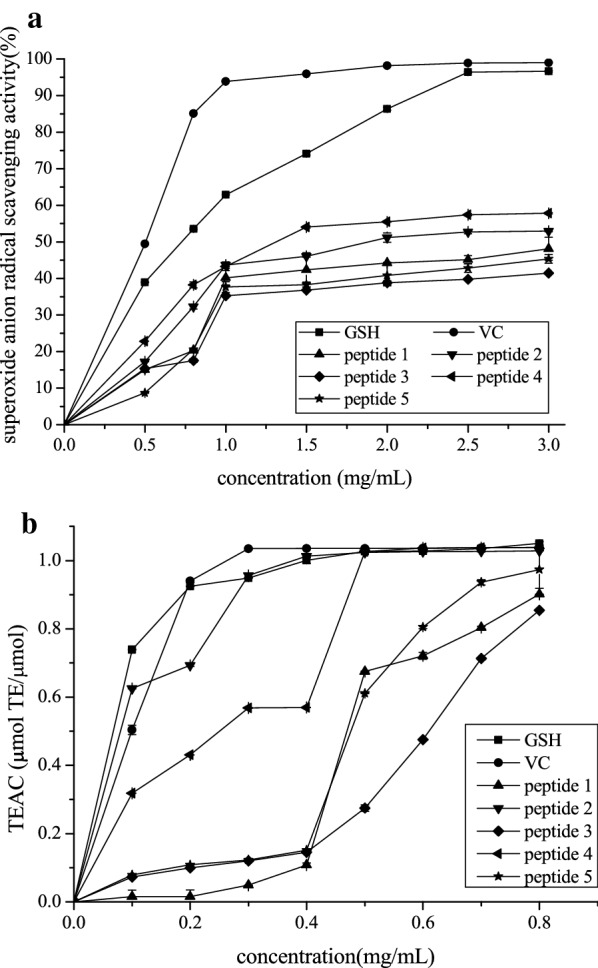
Effect of antioxidant peptide concentration on superoxide anion radical scavenging activity (**a**) and TEAC (**b**) of five synthetic peptides. Data expressed as means ± SD (n = 3)

Superoxide radical is a deleterious free radical that can cause serious damage to human tissues [33]. Jin [34] found that the measurement of scavenging superoxide radical was based on the principle that antioxidants can scavenge superoxide anion radicals generated in the process of pyrogallol autoxidation and inhibit the autoxidation reaction of pyrogallol. As shown in Fig. 5a, the superoxide anion radical scavenging capability of the five synthesized peptides presented a dose-dependent relationship. The antioxidant capacities of the five peptides were all lower than those of ascorbic acid and glutathione (GSH) at 1.0–3.0 mg/mL (*P* < 0.05). However, peptides 2 and 4 exhibited higher superoxide anion radical scavenging activities than those of the three other peptides (*P* < 0.05). Furthermore, the IC50 values of peptides 1, 2, 3, 4, and5 were 2.898, 2.473, 3.662, 2.145, and 3.271 mg/mL, respectively. Thus, peptides 2 and 4 can be considered good superoxide anion radical scavengers.

As shown in Fig. 5b, the TEAC of peptides 2 and 4 were excellent in the concentration range of 0.1–0.8 mg/mL, which were significantly higher than those of the three other peptides (*P* < 0.05). In addition, the TEAC of peptide 2 at 1–0.4 mg/mL was better than that of peptide 4 at 0.5–0.8 mg/mL, and the TEAC of peptides 2 and 4 were substantially the same as those of ascorbic acid and GSH (*P* < 0.05). However, no significant differences were observed among the TEAC of peptide 2 and peptide 4 (*P* > 0.05). The IC50 values of peptides 1, 2, 3, 4, and 5 were 0.763, 0.092, 0.877, 0.205, and 0.750 mg/mL, respectively. Thus, peptides 2 and 4 exhibited better antioxidant capacities than that of the three other peptides.

Peptides 2 and 4 synthesized in this study might be better antioxidants than the other peptides because of their high resistance to superoxide anion radical and high TEACs (Fig. 5) (*P* < 0.05). The peptide concentrations for the determination of superoxide anion radical scavenging activity and TEAC in the stability study (3.5 and 3.6) were chosen to be 1.0 and 0.5 mg/mL, respectively, according to the experimental results.

### Temperature stability

The superoxide anion scavenging capability of each peptide slightly decreased in varying degrees as the temperature increased from 25 °C to 80 °C (*P* > 0.05) but sharply declined when the temperature was raised to 80 °C–100 °C (*P* < 0.05). In addition, no significant differences were found among the superoxide anion radical scavenging activity of the five peptides (*P* > 0.05).

**Fig. 6.**
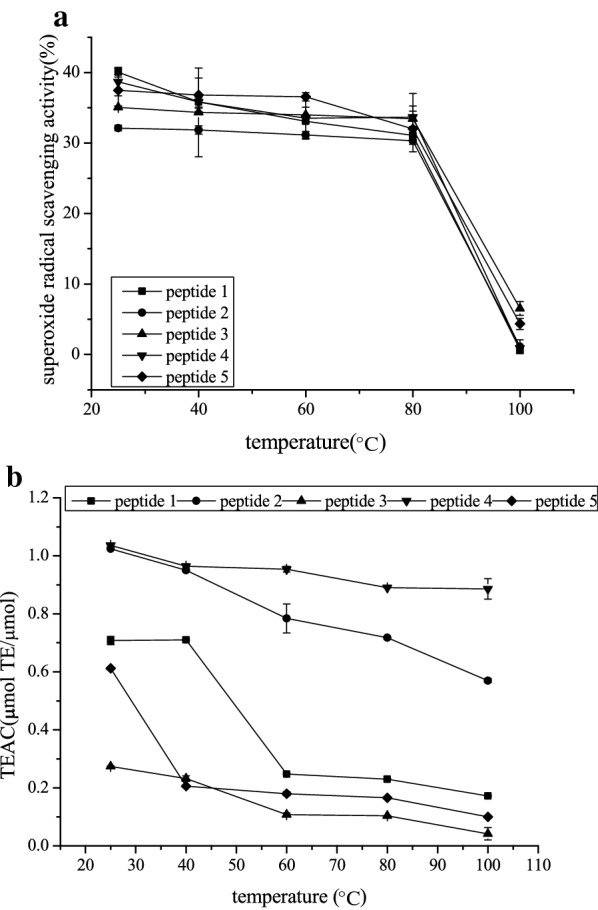
Effect of temperature change on superoxide anion radical scavenging activity (**a**) and TEAC (**b**) of five synthetic peptides. Data expressed as means ± SD (n = 3)

As shown in Fig. 6b, the TEAC of the five peptides all increased in varying degrees and those of peptides 2, 4, and 5 significantly increased (*P* < 0.05) as the temperature rose from 25 °C to 40 °C. However, the TEAC of the five peptides demonstrated downward trends at 60 °C–100 °C. The TEAC of peptides 2 and 4 were significantly higher than those of the three other peptides at 25 °C–100 °C, and the TEAC of peptide 4 was significantly higher than that of peptide 2 (*P* < 0.05) at 60 °C–100 °C. The TEAC trends of the five peptides were consistent with the antioxidant activities of peptides from sardines studied by Jiang et al. [23]. Moreover, these trends were in agreement with data from previous studies, demonstrating that most proteins were denatured at temperatures from 60 °C to 100 °C [35]. These trends may be due to the absence of tertiary and quaternary structures in short-chain and low molecular weight peptides (only those proteins with molecular weight $\ge$ 50 kDa can form quaternary structures). However, these peptides can still form secondary structures, which are the key factors affecting antioxidant activities. Temperatures higher than 60 °C will affect the secondary structure, thus leading to unstable antioxidant activities [36].

Based on these results, peptides 2 and 4 exhibited better temperature stability compared with that of the three other peptides. Thus, peptides 2 and 4 might have good thermal processing stability.

### In vitro digestion stability

**Fig. 7.**
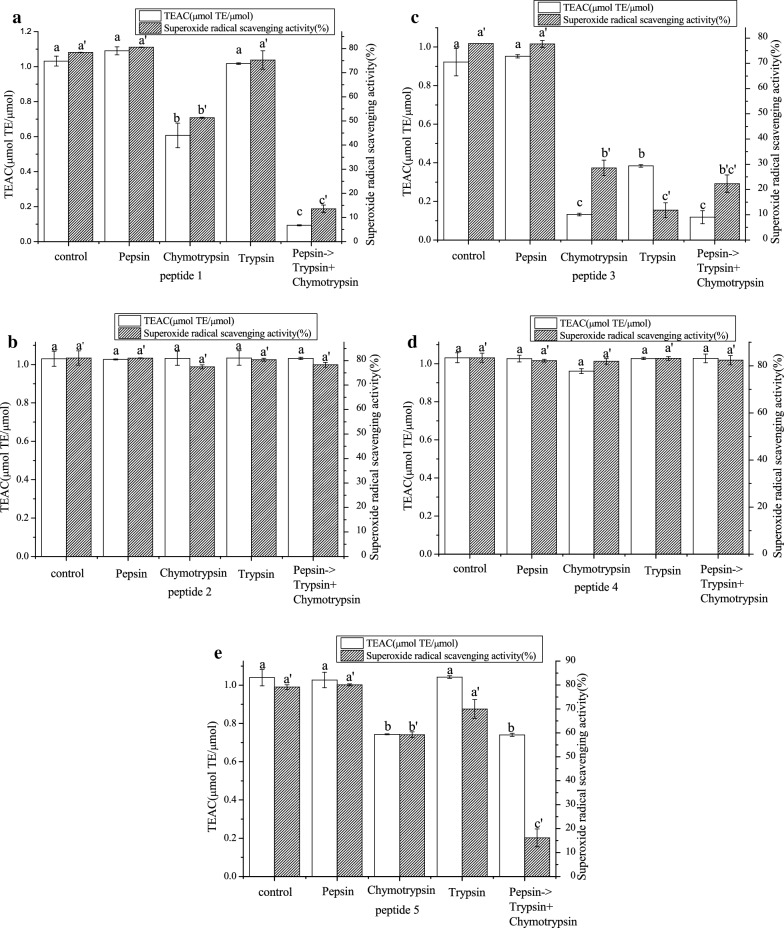
Effects of in vitro digestion on TEAC and superoxide anion radical scavenging activity of five peptide (**a**, **b**, **c**, **d**, **e**). Data expressed as means ± SD (n = 3).Means followed by the same letter were not significantly different according to Duncan’s multiple range test at P < 0.05 level

Figure 7b and d indicated that the TEAC and superoxide anion radical scavenging capabilities of peptides 2 and 4, which were digested by pepsin, trypsin, or chymotrypsin, alone or continuously, remained at the same levels compared with those before digestion (*P* > 0.05). These results indicate that the active structures of antioxidant peptides 2 and 4 were not fully disrupted by intestinal proteases. Therefore, peptides 2 and 4 exhibited improved stability during the in vitro digestion process. The antioxidant activities of peptides 1 (Fig. 7a), 3 (Fig. 7c), and 5 (Fig. 7e) demonstrated insignificant changes (*P* > 0.05) when pepsin acted alone. This phenomenon might be due to the broad specificity of pepsin as an aspartic protease. Pepsin exhibits preferential cleavage of peptides with aromatic or dicarboxylic L-amino acid residues, that is, pepsin preferentially cleaves the C-terminal end of Phe [37]. Pepsin also breaks the peptides into small fragments, thus exposing a large number of internal groups, including some hydrophobic groups, to the environment. These results are consistent with those of Zhu et al. [36].

A previous study showed that the capability of peptides to resist enzymatic degradation partially depends on their amino acid composition [37]. chymotrypsin has relatively broad specificity and cleaves the C-terminal end of hydrophobic residues, especially Phe, Tyr, Trp, and Leu [37]. Peptides 1 (Fig. 7a) and 3 (Fig. 7c) contain Phe, and peptide 5 (Fig. 7e) contains Trp and Phe, which are hydrophobic amino acids. These amino acids were individually or continuously degraded by chymotrypsin, resulting in a significant reduction in the antioxidant activity of peptides 1, 3, and 5 (*P* < 0.05). The same trend was also reported by Zhu [38].

The antioxidant activity of peptide 3 significantly declined (*P* < 0.05) when trypsin acted alone on peptides 1 (Fig. 7a), 3 (Fig. 7c), and 5 (Fig. 7e). Previous studies found that the degradation of basic and aromatic amino acids may decrease the antioxidant activities of peptides [37]. Trypsin is a highly specific endopeptidase that cleaves at the C-terminal end of Arg (basic amino acid) and Lys (aromatic amino acid) residues [39]. The composition of peptide 3 is Arg-Asn-Phe (Fig. 7c) and degraded by trypsin. Thus, the amino acid composition enables the peptide to resist enzymatic degradation. This conclusion is consistent with the results of previous research [39]. Therefore, peptides 2 and 4 exhibited good digestive stability in vitro and simulated gastrointestinal digestion.

## Conclusions

In this study, the five new antioxidant peptides, respectively Cys-Gly-Phe-Pro-Gly-His-Cys, Gln-Ala-Cys, Arg-Asn-Phe, Ser-Ser-Cys, and Trp-Phe, were purified from PWBP hydrolysates and evaluated for their antioxidant capacity and antioxidant stability. The five new peptides all exhibited antioxidant activity, among them Gln-Ala-Cys and Ser-Ser-Cys showing stronger antioxidant capacity. Furthermore, when the temperature was lower than 80 ℃, both Gln-Ala-Cys and Ser-Ser-Cys showed better temperature stability than other three peptides. Compared with other three peptides, Gln-Ala-Cys and Ser-Ser-Cys also showed stronger digestive stability in vitro system by simulating gastrointestinal digestion. The current results provide useful information for PWBP hydrolysates and peptides and new ideas for the production of natural antioxidants instead of synthetic antioxidants. Moreover, these results enable the development and use of purple wheat bran.

## Data Availability

The datasets and samples are available from the authors.

## Abbreviations

PWBP: Purple wheat bran protein
TEAC: Trolox equivalent antioxidant capacity
HPLC: High performance liquid chromatography
BHA: Butylated hydroxy anisole
BHT: Butylated hydroxytoluene
TBHQ: Tert-butylhydroquinone
ROS: Reactive oxygen species
DH: Degree of hydrolysis
